# Tomato Domestication Affects Potential Functional Molecular Pathways of Root-Associated Soil Bacteria

**DOI:** 10.3390/plants10091942

**Published:** 2021-09-17

**Authors:** Lisanne Smulders, Emilio Benítez, Beatriz Moreno, Álvaro López-García, María J. Pozo, Victoria Ferrero, Eduardo de la Peña, Rafael Alcalá Herrera

**Affiliations:** 1 Department of Environmental Protection, Estación Experimental del Zaidín (EEZ), CSIC, C/Profesor Albareda 1, 18008 Granada, Spain; lisanne.smulders@eez.csic.es (L.S.); beatriz.moreno@eez.csic.es (B.M.); rafa.alcala@eez.csic.es (R.A.H.); 2 Department of Soil Microbiology and Symbiotic Systems, Estación Experimental del Zaidín (EEZ), CSIC, C/Profesor Albareda 1, 18008 Granada, Spain; alvaro.lopez@eez.csic.es (Á.L.-G.); mariajose.pozo@eez.csic.es (M.J.P.); 3 Department of Biodiversity and Environmental Management, Campus de Vegazana s/n, University of León, 24071 León, Spain; vferrv@unileon.es; 4 Finca Experimental “La Mayora” CSIC, Instituto de Hortofruticultura Subtropical y Mediterránea (IHSM-UMA), CSIC, Algarrobo, 29750 Málaga, Spain; Eduardo.DeLaPena@ugent.be; 5 Department of Plants and Crops, Faculty of Bioscience Engineering, Ghent University, Coupure Links 653, 9000 Ghent, Belgium

**Keywords:** bacterial functions, co-presence networks, metagenomics, microbial ecology, plant domestication

## Abstract

While it has been well evidenced that plant domestication affects the structure of the root-associated microbiome, there is a poor understanding of how domestication-mediated differences between rhizosphere microorganisms functionally affect microbial ecosystem services. In this study, we explore how domestication influenced functional assembly patterns of bacterial communities in the root-associated soil of 27 tomato accessions through a transect of evolution, from plant ancestors to landraces to modern cultivars. Based on molecular analysis, functional profiles were predicted and co-occurrence networks were constructed based on the identification of co-presences of functional units in the tomato root-associated microbiome. The results revealed differences in eight metabolic pathway categories and highlighted the influence of the host genotype on the potential functions of soil bacterial communities. In general, wild tomatoes differed from modern cultivars and tomato landraces which showed similar values, although all ancestral functional characteristics have been conserved across time. We also found that certain functional groups tended to be more evolutionarily conserved in bacterial communities associated with tomato landraces than those of modern varieties. We hypothesize that the capacity of soil bacteria to provide ecosystem services is affected by agronomic practices linked to the domestication process, particularly those related to the preservation of soil organic matter.

## 1. Introduction

The coevolutionary framework for analyzing interactions between plants and soil microorganisms has mainly been used for organisms involved in rhizosphere processes. Given that rhizosphere microbiomes are part of complex food webs affecting large numbers of nutrients released by the plant, it has been suggested that plants attract and select beneficial microbiomes by first releasing signals and then filtering species [1,2]. Rhizopshere microbiota are well known to play a critical role in both the adaptation of plants to the environment, but also contribute to a wide range of essential ecosystem services, such as carbon and nutrient cycling, plant growth promotion, soil structure stability, food web interactions and soil-atmosphere gas exchange, which ultimately affect soil productivity and sustainability [3].

In addition to plant genetics and developmental stage [4,5], other factors including soil management, agronomic practices, pathogen presence, soil pH, nutrient content, and moisture, have been suggested to affect root microbial community structure [6,7,8]. However, the question of how the host and its environment regulate microbiome assembly and co-occurrence in plant species has not been addressed yet. This is of particular interest for crops in the context of plant–soil feedback, where plants can change soil biology and chemistry in ways that could affect subsequent plant growth [9].

Crop genetic diversity is usually reduced during plant domestication, which is associated with the selection of certain morphological traits such as root architecture and exudate composition, leading to striking differences between crops and their wild relatives [10,11]. Therefore, domestication is expected to have a direct impact on the type and diversity of below-ground microorganisms [9]. Indeed, domestication and genetic selection have progressively differentiated the microbiota of modern crops from those of their wild progenitors. It has also been postulated that crops are more likely to display negative feedbacks as compared to wild relatives, as domestication potentially disrupted beneficial rhizosphere associations [12]. Previous studies of cultivated plants evidenced differences between bacteria associated with differing plant genotypes such as wheat (*Triticum aestivum* L.), rice (*Oryza sativa* L.), barley (*Hordeum vulgare* L.) and tomato (*Solanum lycopersicum* Mill.) [13,14,15], suggesting that traits selected during domestication could have a significant influence on rhizosphere microbiota composition.

Although the structure of root-associated microbial communities is widely accepted to depend, to a greater or lesser degree, on the plant genotype, little is known about whether domestication-mediated differences between rhizosphere microorganisms functionally affect microbial ecosystem services. In this scenario, an evaluation of functional soil microbial genes could help to determine the effect of domestication on functional redundancy or co-occurrence of basic metabolic capacity in the rhizospheres of crop varieties and their wild ancestors [16]. This is essential to identify agricultural practices that resulted in reduced trade-offs between agricultural productivity and the provision of ecosystem services.

This study aims to explore how plant domestication influences the assembly patterns of soil microbial communities by metagenomic analysis of bacterial communities and predicted functions in the rhizosphere of different tomato varieties along a domestication gradient.

## 2. Results

### 2.1. Bacterial Community Structure

**Figure 1:**
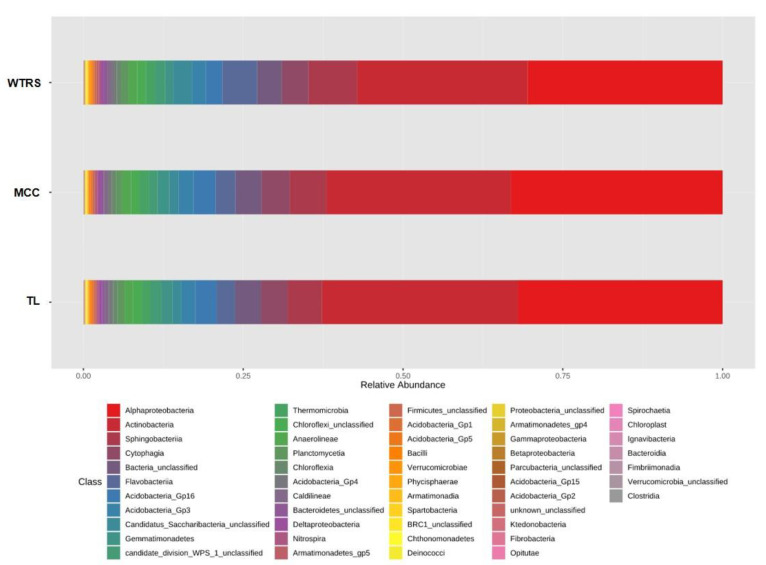
Relative abundance of bacteria of tomato rhizosphere soils. WTRS: wild tomato related species; TL: tomato landraces; MCC: modern commercial cultivars.

Figure 1 shows the relative bacterial abundance of the tomato root-associated soils based on the 16S rRNA gene. Two main bacterial classes, Alphaproteobacteria and Actinobateria, dominated the total bacterial community with no differences observed between plant groups. Minority phyla such as Acidobacteria (*F* = 7.152, *p* = 0.002) and Gemmatimonadetes (*F* = 4.720, *p* = 0.013) were significantly less represented in the rhizosphere of wild tomato species than in tomato landraces and modern commercial cultivars. At the family level, the relative abundance of the *Gemmatimonadaceae* (*F* = 4.133, *p* = 0.022), *Microbacteriaceae* (*F* = 5.419, *p* = 0.007), and *Streptomycetaceae* (*F* = 4.752, *p* = 0.022) families decreased, while *Sphingomonadaceae* (*F* = 7.887, *p* = 0.001) increased in wild tomato relatives. Again, no differences between tomato landraces and modern commercial cultivars were detected.

The relative abundances of Acidobacteria_Gp16_unclassified (*F* = 3.701, *p* = 0.031), *Hyphomicrobiaceae* (*F* = 6.736, *p* = 0.002), and *Nocardioidaceae* (*F* = 4.179, *p* = 0.021) were different between wild and commercial cultivars, while landraces had intermediate values, generally not differing from the other two groups.

**Figure 2:**
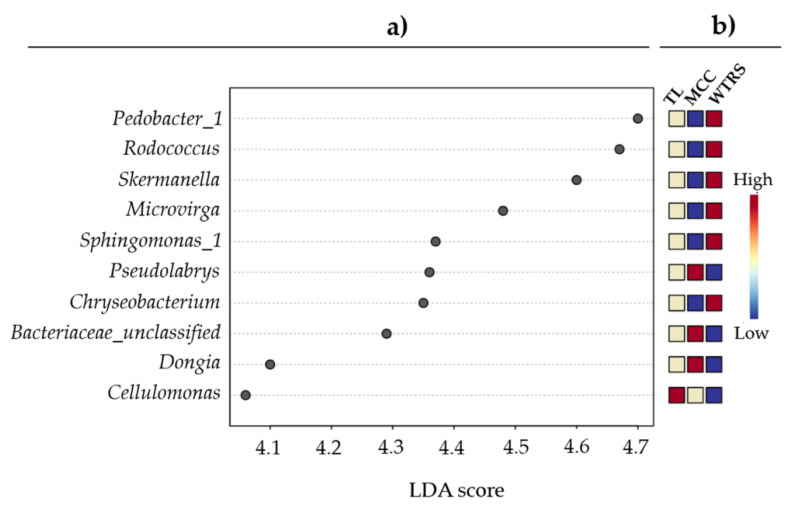
(**a**) Linear discriminant analysis (LDA) scores and, (**b**) heatmap from blue (low) via white to red (high) of genus relative abundances in root-associated soil of wild tomato related species (WTRS), tomato landraces (TL), and modern commercial cultivars (MCC).

Linear discriminant analysis (LDA) at the genus level showed *Pedobacter* (*Sphingobacteriaceae*), *Rodococcus*, *Skermanella* and the proteobacterium *Microvirga* to be mainly responsible for the differences between the three tomato clusters (Figure 2). In addition, minor changes in bacterial diversity were observed at the OTU level (Table 1), as indicated by a significant decrease in the evenness of crop wild relatives (*F* = 6.623, *p* = 0.003).

### 2.2. Bacterial Community Functional Analysis

We used metagenomics analysis to predict the functional potential of the bacterial community and to explore associated metabolic pathway networks using Kyoto Encyclopedia of Gene and Genome (KEGG) clusters.

At the level of functional units of gene sets, all tomato varieties shared all the 181 predicted functions related to soil bacteria (Table S1). However, 68 of them differed among tomato domestication types (Table 2). In general, wild tomatoes differed from modern cultivars and tomato landraces that usually showed similar values, but generally tomato landraces had intermediate values between modern cultivars and wild relatives. For example, the levels of the aromatic degradation metabolic pathway category, except for module M00541 (benzoyl-CoA degradation), tended to be significantly higher in bacteria growing in wild tomato accessions, indicating that tomato landraces drive bacterial communities with similar levels of predicted functions as modern commercial cultivars. Similarly, while the values for the metabolic categories of nitrogen, sulfur, cofactor/vitamin and purine were decreased in modern cultivars with respect to wild varieties, no differences between these wild and landrace cultivars were detected. By contrast, lipopolysaccharide and lipid metabolic pathway levels were clearly higher in both landrace and modern cultivars with respect to their wild relatives.

Amino acid metabolism pathways exhibited no clear tendency, although in modern commercial cultivars, cysteine and methionine pathway levels were higher and those of other amino acid pathways were lower. A similar variable pattern was observed with respect to both central carbohydrate and other carbohydrate metabolic pathways in the category of carbohydrate metabolism.

Finally, a marked increase in the carbon fixation and methane metabolic subfunctions and in the metabolic pathway categories glycan metabolism and lipid metabolism, respectively, was observed in the modern commercial cultivars.

### 2.3. Functional Networks of KEGG Orthologous Groups

**Figure 3:**
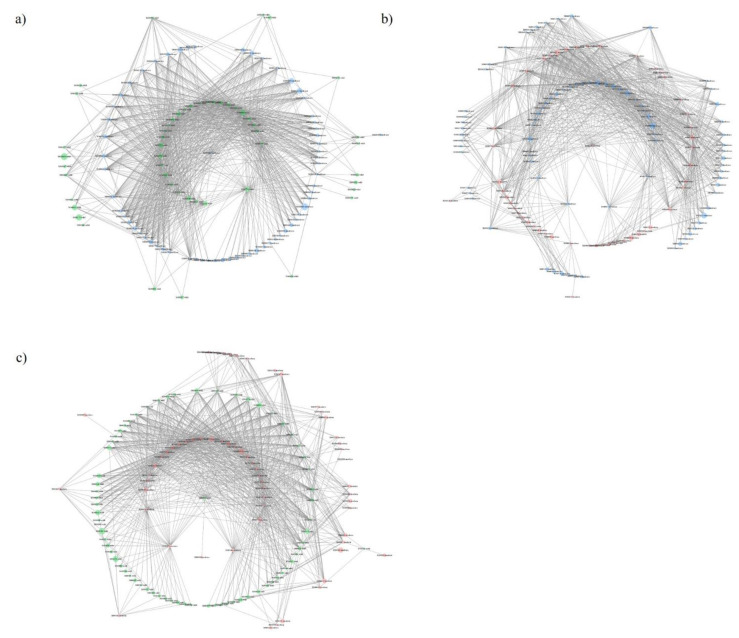
Co-presence network for the couples: (**a**) tomato landraces:wild tomato related species, (**b**) modern commercial cultivars:tomato landraces, (**c**) modern commercial cultivars:wild tomato related species. Node sizes reflect average relative abundance of each KEGG module. The line thickness is proportional to the edge weight. Node colors: green for wild tomato related species, blue for tomato landraces and red for modern commercial cultivars.

Figure 3 and Table 3 show the co-presence networks and the topological properties of functional networks, respectively, for the modern:wild, landraces:wild and modern:landraces pairs. An increase in the average number of neighbors and a decrease in the characteristic path length were found in landraces:wild pairs (Table 3). Additionally, an increase in the network radius and diameter were detected in the pair modern:landraces. Finally, the pair modern:wild showed the largest number of KEGG-module nodes and the largest number of edges or inter-node connections in the network. The clustering coefficient, which reflects the tendency of organisms to form relatively high-density clusters, was zero. Co-occurrence networks are generated by connecting pairs of terms using a set of criteria defining co-occurrence. These networks connect across, rather than between, nodes. Every node, in which none of whose neighbors connect to each other, has a clustering coefficient of zero.

**Figure 4:**
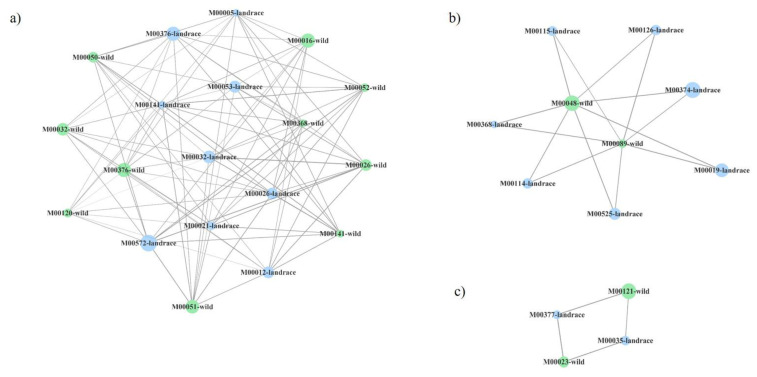
Three (**a**–**c**) most connected clusters in co-presence networks for the couple tomato landraces (blue):wild tomato related species (green). Node sizes reflect average relative abundance of each KEGG module. The line thickness is proportional to the edge weight.

**Figure 5:**
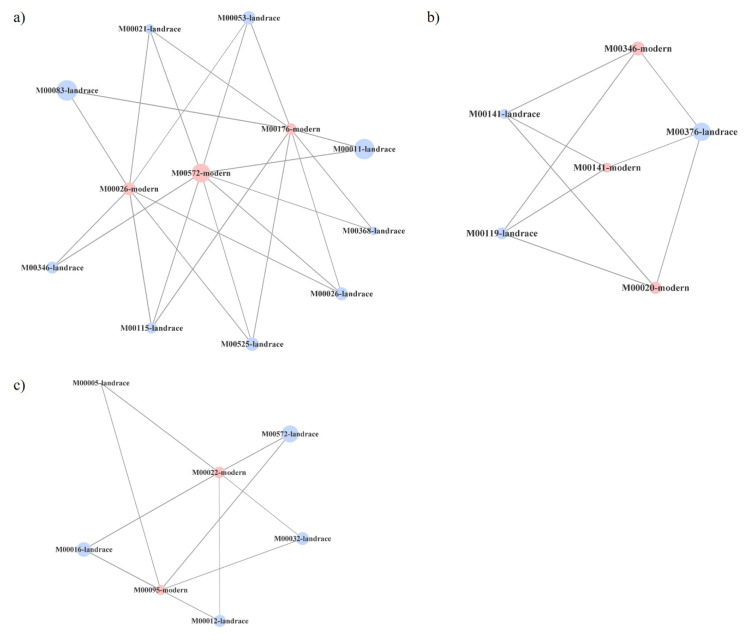
Three (**a**–**c**) most connected clusters in co-presence networks for the couple modern commercial cultivars (red):tomato landraces (blue). Node sizes reflect average relative abundance of each KEGG module. The line thickness is proportional to the edge weight.

**Figure 6:**
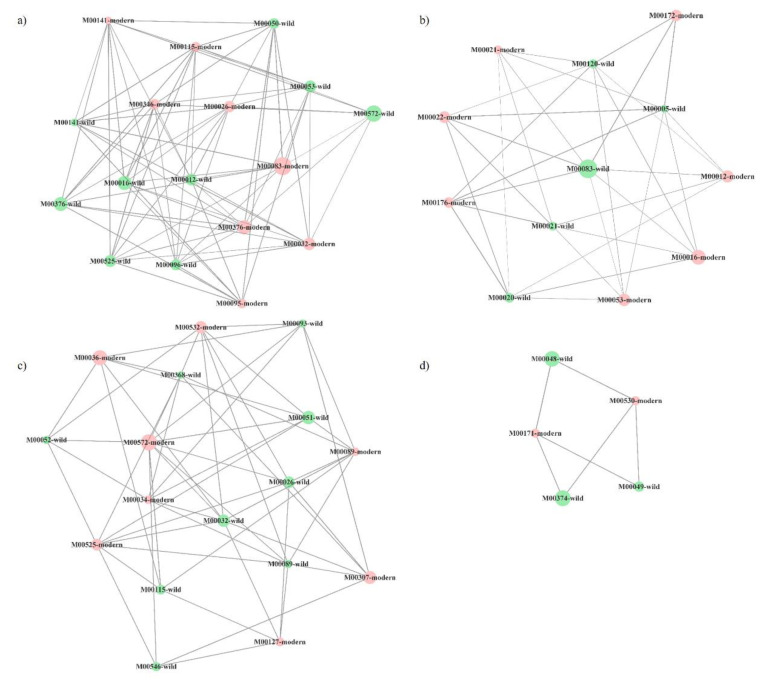
Four (**a**–**d**) most connected clusters in co-presence networks for the couple modern commercial cultivars (red):wild tomato relates species (green). Node sizes reflect average relative abundance of each KEGG module. The line thickness is proportional to the edge weight.

Highly connected clusters were retrieved for every network, four for the pair modern:wild and three for the other two pairs (Figure 4, Figure 5 and Figure 6). On a closer analysis, we detected some links in highly connected clusters. Thus, bacterial functional units M00026 (histidine biosynthesis, PRPP => histidine), M00032 (lysine degradation, lysine => saccharopine => acetoacetyl-CoA), M00141 (C1-unit interconversion) and M00376 (3-hydroxypropionate bi-cycle) were highly connected in the soil of tomato landraces and wild relatives (Figure 4a), whereas modern varieties and wild relatives were connected by modules M00141, M00376, M00021 (cysteine biosynthesis, serine => cysteine) and M00089 (triacylglycerol biosynthesis) (Figure 5a–c). Finally, modules M00026 and M00141 were highly represented in modern varieties and tomato landraces (Figure 6a,b).

## 3. Discussion

Root traits selected during domestication were previously suggested to have a significant influence on the composition of the rhizosphere microbiome [13,17]. We found similar core bacterial microbiome members in tomato landraces and modern commercial cultivars, but detected small, though significant, differences in bacterial communities associated with both their rhizospheres and those of wild tomato relatives (Figure 1).

At family level, *Gemmatimonadaceae* (phylum Actinobacteria), *Microbacteriaceae* and *Streptomycetaceae* (Gemmatimonadetes) were represented less in the rhizosphere of wild tomato related species. At genera level, domestication gradually reduces the presence of the ubiquitous soil bacterium *Pedobacter*, the aromatic substrate metabolizer Rhodococcus and the alphaproteobacteria *Skermanella* and *Microvirga*, the latter considered a symbiotic nitrogen-fixing bacterium.

Previous studies highlighted the effect of plant species on the microbial composition and OTU abundance of the rhizosphere microbiome [5,18]. Domesticated crops often have shallow roots and shifts in traits such as leaf size and root architecture. Changes in these morphological traits results in increased litter quality, lower C:N ratio and root exudate composition, which could influence microbial community composition [2,9,19,20]. In this study, bacterial diversity at the OTU level was found to remain virtually unchanged along the domestication gradient, although evenness levels were significantly lower in the rhizosphere of tomato wild relatives. Evenness refers to the similarity of OTU frequencies in bacterial populations. Even though species evenness and richness are complementary, no differences were observed in the latter; the number of soil bacterial phyla recruited by wild type crops was similar to other tomatoes. Nevertheless, evenness does not necessarily translate into optimal diversity; ecosystem functions at the bacterial community level are more important than the bacterial species. As several species in an ecosystem may fulfill a similar function (redundancy), their even distribution is not essential as long as the function itself remains active. However, a more even species distribution within a bacterial community is assumed to make the ecosystem more resilient, as the risk of losing an essential component of the functional network would be much lower.

Using metagenomic analysis, the functional potential of the bacterial community was predicted and the associated metabolic pathway network explored (Table 2). The levels of the global metabolic pathway for aromatic degradation were significantly higher in bacteria associated to accessions of tomato wild relatives. The modules belonging to this pathway catalyze reactions involving various polyphenols such as catechol. Humification is known to involve biotic and abiotic transformations of soil litter layer materials into mature humic substances, where catechol and o-quinones derived from biotic activity in humic substance synthesis play a fundamental role [21]. In addition, the increase in catechol promotes the formation of humic substances through abiotic reactions in the catechol–Maillard system [22]. Thus, the observed decrease in the degradation of aromatic compounds to catechol indicates a loss of degradation capacity due to cultivation. Organic matter and humic substances play an important role in improving soil fertility and structure, water retention capacity and C sequestration in the environment [23], which diminishes along the domestication gradient. Another possible hypothesis is that plants affect microbial populations, and changes in environmental conditions, soils and cultivation techniques—with the gradual abandonment of organic materials in favor of agrochemicals—could reduce the degradation capacity of recalcitrant organic compounds associated with domestication and breeding. On the other hand, with respect to the carbon cycle, organic C taken up by microorganisms is partitioned into growth, metabolite excretion, and respiration [24]. We detected an increase in the Krebs cycle of wild tomato related species below-ground. After incorporation into the bacterial biomass, C is usually converted into stable organic matter or decomposed and respired as CO_2_ depending on the chemical recalcitrance and degree of protection of the organic matter [25].

In this context, it has been suggested that crop wild relatives establish beneficial interactions with microbes more frequently than domesticated cultivars [26]. Given the abandonment of some agricultural practices related to exogenous organic matter inputs and the preservation of endogenous C, a concomitant loss of bacterial functions dealing with recalcitrant organic matter has been occurring for many years. It has also been evidenced that agronomic practices, such as tillage, irrigation and the use of other inputs such as pesticides and fertilizers influence the below-ground diversity and functions of soil microbes [27]. We therefore postulate that a loss in bacterial functions related to soil organic matter preservation occurs during tomato domestication.

A similar trend was detected in metabolic pathways related to biochemical cycles, such as the reduction in nitrates and sulphates and the formation of urea from purine metabolism. The decrease in these pathways that play a key role in plant growth could be attributed to the domestication process, or more precisely, to the emergence of modern commercial cultivars. Similar to the observations in the C-cycle, the increasing use of agrochemicals in modern agriculture may, in some way, be connected to the reduction on metabolic pathway levels caused by certain biochemical cycles.

Carbon fixation was more common in bacteria associated with modern commercial cultivars. This important process in soil carbon cycling is carried out by CO_2_-fixing and CO-oxidizing bacteria and can reduce atmospheric CO_2_ concentrations, thus indirectly mitigating global warming [24,28,29]. However, as no differences in the synthesis of ribulose 5 phosphate, an intermediary in the carbon fixation Calvin cycle, can be attributed to domestication, it is not possible to draw a clear picture of the effects of domestication on this ecosystem service.

On the other hand, pathways such as fatty acid and jasmonic acid biosynthesis were more commonly found in the rhizosphere of modern and landrace varieties. Fatty acids are involved in multiple functions, ranging from cell membrane constituents to cell signaling. Fatty acids have been used as indices of soil quality and even to describe food web connections [30], thus, positive feedback compared with their wild ancestors could be attributed to tomato crops. Jasmonic acid (JA) and its derivatives (collectively known as jasmonates) play an important role in regulating plant defenses against biotic stresses, and facilitating beneficial interactions between plants and microbes in the root zone [31,32]. JA signaling has been suggested to have evolved during land colonization by plants exposed to new biotic and abiotic stresses [33], and symbiotic relationships with microbes, including plant growth promoting bacteria and mycorrhizal fungi. Moreover, microbe induced systemic resistance to pathogens and pests involve JA signaling [34,35]. However, although JA production by bacteria and fungi in soil has been reported [36], its impact on plant–microbiome interactions remains unclear. Finally, regarding signalling, we detected a significant increase in the biosynthesis of gamma-aminobutyric acid (GABA) in wild tomato species compared to the groups that included cultivars. GABA is involved in inter-bacterial communication and interactions between bacteria and their host [37]. Furthermore, GABA production has been associated with bacterial overcoming of environmental stress [38].

Overall, these findings highlight the influence of tomato domestication on some molecular pathways of the associated soil bacteria, although all ancestral functional characteristics of bacteria have been conserved across time. However, we wonder whether there is a pattern of bacterial functional abundance associated with the tomato soil related to the domestication degree. To shed some light on this point, we calculated interactions between functional units of gene sets in metabolic pathways, which may help to address the question of how microbial genes work together to support specific microbiome functions [39]. In this study, we assessed pairwise relationships between bacterial functional units based on metagenomic sequencing of bacteria growing on tomato plants along a domestication gradient The highest connectance levels in bacterial communities were found in landraces:wild pairs due to an increase in network density as measured by the higher average number of connections established expressed by the average number of neighbors (Table 3). In addition, the increased connectance in the landraces:wild pairing with respect to the other two pairs was related to the decrease in the characteristic path length, defined as the average number of steps along the shortest paths for all possible pairs of network nodes. These changes suggest an intensification of microbial connectance relative to the pairs modern:landraces and modern:wild. Finally, an increase in the pair modern:landraces regarding the network radius and diameter measuring the longest of all the shortest calculated paths in the network, suggests a decrease in module-pathway connectance.

Highly connected clusters, or sets of nodes most of which are connected with one another, were then explored (Figure 4, Figure 5 and Figure 6). Again, the highest connectance was detected for the pair landraces:wild varieties and nodes representing the same module in the two different types of tomato were recovered in a single cluster. For the rest of the pairs, even if they shared the same number of common modules, they were recovered in two or three different clusters. Overall, the above results suggest that certain functional groups such as the synthesis of certain amino acids or carbohydrate metabolism tend to be more evolutionarily conserved in bacterial communities associated with tomato landraces than those of modern varieties. However, we also found that most of the metabolic routes of bacteria associated to either landraces or modern cultivars with those associated to their ancestors were different. In this scenario, a possible process of divergent evolution in tomato lines, that is, the process by which groups of the same common ancestor evolve and accumulate differences in response to changes in both environmental conditions and biotic factors, could be debated. Nevertheless, further investigation is needed to clarify how tomato domestication has driven specific bacterial functions in root-associated soil.

## 4. Materials and Methods

### 4.1. Field Experiment

Seeds of 27 *Solanum lycopersicum* Mill., *S. habrochaites* and *S. pimpinellifolium* accessions were selected from La Mayora Institute of Subtropical and Mediterranean Horticulture (IHSM-UMA-CSIC) germplasm bank. Seeds were germinated and ten one-month-old seedlings per variety (n = 270) were randomly sown in an experimental field of La Mayora IHSM (Málaga, Spain; 36.77° N, 4.04° W),) on 19th April 2018 in a Eutric Regosol soil [40]. They were grown until 16th July 2018. Just after transplanting, plants were watered with 15:15:15 solution (15% nitrogen, 15% phosphorus and 15% potassium) during 30 min adding-up a volume of 4 l per plant. During the course of the experiment, watering consisted of 30 min of water twice in a week (Mondays and Fridays) [41]. At harvest, the soil attached to the main and secondary roots was taken by shaking, placed in separate plastic bags, and kept at 4 °C. Then, samples from each variety were pooled, ground together using a mortar and pestle, sieved twice (2-mm mesh) and immediately stored at −20 °C until molecular analyses were performed.

In this study, cluster assays of the 27 tomato accessions, based on their degree of domestication, were carried out: (1) wild tomato species (accessions NR0407, NR1021, NR0136, NR0699, NR0937), (2) tomato landraces (accessions NR0025, NR0006, NR0044, NR0213, NR0275, NR0237, NR0469, NR0166, NR0063, NR0705, NR0612), and (3) modern commercial cultivars (accessions ABL104, ANL101, NR0071, NR0816, NR0080, NR1080, NR0504. COM1, COM2, COM3 and COM4 cultivars, which are protected under plant variety rights, have no accession number).

### 4.2. Chemical Characteristics of the Soil

Air-dried rhizosphere soil samples were used to determinate chemical properties. Total N and SOC were determined with the aid of the Leco-TruSpec CN elemental analyzer (LECO Corp., St Joseph, MI, USA). Total mineral content was determined by the digestion method with HNO3 65%:HCl 35% (1:3; v:v) followed by analysis using inductively coupled plasma optical emission spectrometry (ICP-OES) (ICP 720-ES, Agilent, Santa Clara, CA, USA). Detailed information on soil characteristics is given in Supplementary Material Table S2.

### 4.3. Molecular Analyses of Soil Bacteria

DNA was extracted from eight 1 g aliquots for each root-associated soil sample using the bead-beating method with the aid of a PowerSoil^®^ DNA Isolation Kit (MoBio Laboratories, Solana Beach, CA, USA) according to the manufacturer’s instructions. For each variety, two replicates were prepared by pooling four extractions and concentrating them at 35 °C to a final volume of 20 μL using a Savant Speedvac^®^ concentrator (Fisher Scientific, Madrid, Spain). The V3-V4 hypervariable regions (ProV3V4 primers 5′ CCTACGGGNBGCASCAG 3′ and 5′ GACTACNVGGGTATCTAATCC 3′ [42,43]) of the 16S rRNA gene were used to characterize the bacterial communities of the two replicates per sample using the Illumina MiSeq platform (2 × 250 nucleotide paired-end protocol) at the genomic facilities of the López-Neyra Institute of Parasitology and Biomedicine (IPBLN-CSIC). Blockers were used to minimize amplification of mitochondria and chloroplasts. Raw sequences were preprocessed using the SEED2 platform [44] by first merging forward and reverse sequences. Quality filtering excluded sequences containing ambiguous bases (N) and those with a quality score of less than 30. Primers were removed and sequences trimmed to 400 bp length. The sequences were then clustered using the UPARSE method [45]: Operational Taxonomic Unit (OTU) radius set to 3% and sequence similarity to 97%. Singletons and chimeric sequences were removed. Taxonomic assignment of OTUs was performed using the classify.seqs algorithm in Mothur software (University of Michigan, Detroit, MI, USA) against the SILVA v132 database, after which no archaea were detected in the samples [46,47]. An abundance sample x OTU matrix was generated using OTU reads as a proxy of abundance using the Marker Data Profiling module in the MicrobiomeAnalyst tool (https://www.microbiomeanalyst.ca/ accessed on 2 August 2021). The most abundant sequence per OTU was selected as representative. Rarefaction curves were visualized using MicrobiomeAnalyst to confirm that all samples reached a plateau [48,49].

### 4.4. Predictive Metagenomics Profiling

To determine the potential functional metabolic capabilities of soil bacterial communities, we used Tax4fun, an open-source R package, which predicts the functional capabilities of these communities based on 16S datasets. Tax4Fun is applicable to output obtained from the SILVAngs web server [50]. Tax4fun was implemented in Shotgun Data Profiling (SDP) module of MicrobiomeAnalyst to predict functional pathways based on Kyoto Encyclopedia of Gene and Genome (KEGG, https://www.kegg.jp/ accessed on 2 August 2021) annotations [51,52]. KEGG functional annotations were based on modules, i.e., functional units of gene sets in the KEGG metabolic pathways database that can be linked to specific metabolic capacities and other phenotypic features [48].

### 4.5. Functional Networks

Similarities on functional profiles across tomato types were studied by looking for correlations in the abundance of modules. CoNet plug-in method [53] in Cytoscape software v.3.8.2 [54] was used to visualize these relationships by building co-occurrence networks. Thus, two nodes representing the same module in different tomato types should be connected in the case that both tomatoes have a similar pattern of abundance for that module. Thus, building networks by tomato type pairs gives an idea of the conservation of modules across domestication (i.e., the number of links between the same module in different tomato types). Co-occurrence networks were constructed based on the identification of significant positive associations, that is, co-presences of functional units in the tomato root-associated microbiome. Due to the different number of samples/tomato varieties in each domestication type, for arranging the construction of network, the number of samples in each domestication type was adjusted to the tomato type with the least number. The selection and order of samples was arranged randomly. This analysis was repeated 5 times by shuffling the input sample order to avoid spurious results. To run the analysis, KEGG modules having less than 20 reads were discarded from the analyses. KEGG module abundance was normalized by sample. A total of 2000 permutations were set up by keeping edge number constant. The significance of co-presences were evaluated by a combination of Spearman and Pearson correlations and Bray–Curtis dissimilarity (see e.g., [52,55], corrected for multiple testing using Bonferroni). Finally, the MCODE Cytoscape plugin [56] with default settings was then used to detect highly connected network modules. Only modules with an MCODE score greater than 2.0 were retained for analysis [39].

### 4.6. Statistical Analyses

OTU abundance information was normalized to the abundance value of the sample with the least number of sequences. Alpha diversity indices generated by SEED2 were used to compare bacterial richness and diversity in tomato accessions. Statistically significant differences in alpha diversity, the bacterial composition of the group of tomato varieties and predictive metagenomics profiling data were evaluated using generalized lineal model (GLM) with degree of domestication as fixed factor. We checked fixed factors for significance with Wald test from car package [57] and multiple comparisons between levels of the fixed factor were tested using Tukey’s test with the package lsmeans and emmeans [58]. For each model, residuals were examined for model validation. Beta diversity, or species complexity differences between groups of tomato varieties, was determined by linear discriminant analysis (LDA) effect size (LEfSe) using the MicrobiomeAnalyst web server. Taxa with an LDA score > 4 were considered important biomarkers of each group given that a *p* value < 0.05 indicates significant differences between groups. Data were analyzed using R version 3.6.3 [59] and R Studio version 1.1.456 [60].

## 5. Conclusions

In our study we found that core bacterial microbiome is similar between tomato landraces and modern commercial cultivars with small differences with wild tomato. These findings highlight the influence of the host genotype on the potential functions of soil bacterial communities. Furthermore, we found that differences in eight biological metabolic pathways between wild tomatoes compared with tomato landraces and modern commercial. Thus, we conclude that all ancestral functional characteristics of bacteria have been conserved across time. In the light of these results, it becomes apparent that the capacity of soil bacteria to provide ecosystem services is affected by agronomic practices linked to the domestication process, particularly those related to the preservation of soil organic matter. We also assayed the relationships between functional units of bacteria growing on tomato plants along a domestication gradient, finding the highest levels of connection between bacterial communities driven by tomato landraces and their wild ancestors.

## Figures and Tables

**Table 1:** Richness estimates and diversity indices (means ± SE) for 16S rRNA libraries of tomato rhizosphere soils. Different letters indicate a significant difference among tomato varieties (*p* < 0.05, ANOVA, Dunn’s post hoc-Bonferroni corrected *p* values) when exist. WTRS: wild tomato related species; TL: tomato landraces; MCC: modern commercial cultivars.

	WTRS	TL	MCC
Shannon–Wiener Diversity Index	5.79 ± 0.26	6.23 ± 0.06	6.18 ± 0.09
Shannon Entropy	8.35 ± 0.37	8.98 ± 0.08	8.92 ± 0.12
Species Richness	3260 ± 346	3727 ± 155	3471 ± 231
Total Abundance	52,838 ± 3338	59,144 ± 1702	57,100 ± 2964
Simpson Diversity Index	0.031 ± 0.013	0.010 ± 0.001	0.011 ± 0.002
Evenness	0.719 ± 0.021b	0.759 ± 0.004a	0.764 ± 0.005a
Chao-1	4453 ± 450	4983 ± 241	4619 ± 329

**Table 2:** Functional units of gene sets in metabolic pathways (KEGG modules) of tomato rhizosphere soils differentially represented among tomato varieties. Different letters indicate a significant difference among tomato varieties (ANOVA, Dunn’s post hoc-Bonferroni corrected *p* values). WTRS: wild tomato related species; TL: tomato landraces; MCC: modern commercial cultivars.

*Pathway Modules*		WTRS	TL	MCC	*p*-Value
**Amino acid metabolism; Arginine and proline metabolism**	M00015_Proline biosynthesis, glutamate =>proline	1656a	1609b	1626b	0.02086
	M00023_Tryptophan biosynthesis, chorismate => tryptophan	3173b	3234a	3205a	0.008801
	M00037_Melatonin biosynthesis, tryptophan => serotonin => melatonin	69b	77a	74ab	0.04182
	M00040_Tyrosine biosynthesis, chorismate => arogenate => tyrosine	508a	464b	467b	5.579 × 10^−6^
	M00042_Catecholamine biosynthesis, tyrosine => dopamine => noradrenaline => adrenaline	95b	106a	104ab	0.02289
	M00533_Homoprotocatechuate degradation, homoprotocatechuate => 2-oxohept-3-enedioate	491a	474b	473ab	0.04715
**Amino acid metabolism; Aromatic amino acid metabolism**	M00545_Trans-cinnamate degradation, trans-cinnamate => acetyl-CoA	1048a	1004b	1014b	0.001853
**Amino acid metabolism; Branched-chain amino acid metabolism**	M00036_Leucine degradation, leucine => acetoacetate + acetyl-CoA	7057a	6938b	6874b	6.597 × 10^−5^
**Amino acid metabolism; Cysteine and methionine metabolism**	M00017_Methionine biosynthesis, apartate => homoserine => methionine	5393b	5464a	5422a	0.006857
	M00035_Methionine degradation	2031b	2101a	2065b	6.836 × 10^−5^
	M00338_Cysteine biosynthesis, homocysteine + serine => cysteine	233c	276a	257b	9.38 × 10^−11^
**Amino acid metabolism; Lysine metabolism**	M00031_Lysine biosynthesis, mediated by LysW, 2-aminoadipate => lysine	81b	106a	98b	1.069 × 10^−5^
**Amino acid metabolism;** **Other amino acid metabolism**	M00118_Glutathione biosynthesis, glutamate => glutathione	944a	876b	880b	7.781 × 10^−8^
	M00027_GABA (gamma-Aminobutyrate) shunt	2018a	1942b	1921b	1.037 × 10^−5^
**Amino acid metabolism; Polyamine biosynthesis**	M00133_Polyamine biosynthesis, arginine => agmatine => putrescine => spermidine	865b	895a	879ab	0.01599
	M00134_Polyamine biosynthesis, arginine => ornithine => putrescine	872a	838b	841b	0.008463
	M00136_GABA biosynthesis, prokaryotes, putrescine => GABA	677a	620b	636b	6.265 × 10^−9^
**Amino acid metabolism; Serine and threonine metabolism**	M00555_Betaine biosynthesis, choline => betaine	1473a	1377b	1383b	4.206 × 10^−11^
**Carbohydrate metabolism; Central carbohydrate metabolism**	M00006_Pentose phosphate pathway, oxidative phase, glucose 6P => ribulose 5P	1535ab	1523b	1546a	0.04953
	M00077_Chondroitin sulfate degradation	105b	118a	123a	6.408 × 10^−5^
	M00008_Entner–Doudoroff pathway, glucose-6P => glyceraldehyde-3P + pyruvate	1993a	1905c	1928b	2.838 × 10^−6^
	M00009_Citrate cycle (TCA cycle, Krebs cycle)	12,529a	12,667b	12,563a	0.0001383
	M00011_Citrate cycle, second carbon oxidation, 2-oxoglutarate => oxaloacetate	9185b	9286a	9207b	0.001811
	M00003_Gluconeogenesis, oxaloacetate => fructose-6P	5474b	5544a	5498b	0.008293
	M00633_Semi-phosphorylative Entner–Doudoroff pathway, gluconate/galactonate => glycerate-3P	85b	91ab	95a	0.03837
**Carbohydrate metabolism; Other carbohydrate metabolism**	M00061_D-Glucuronate degradation, D-glucuronate => pyruvate + D-glyceraldehyde 3P	1694a	1654b	1680ab	0.01455
	M00081_Pectin degradation	113b	129a	132a	6.318 × 10^−5^
	M00114_Ascorbate biosynthesis, plants, glucose-6P => ascorbate	2958ab	2995a	2949b	0.0271
	M00131_Inositol phosphate metabolism, Ins(1,3,4,5)P4 => Ins(1,3,4)P3 => myo-inositol	1003a	969b	968ab	0.02686
	M00550_Ascorbate degradation, ascorbate => D-xylulose-5P	27a	19b	18b	1.195 × 10^−5^
	M00554_Nucleotide sugar biosynthesis, galactose => UDP-galactose	199b	207ab	217a	0.005133
	M00565_Trehalose biosynthesis, D-glucose 1P => trehalose	3380b	3603a	3666a	2.2 × 10^−16^
**Energy metabolism;** **Carbon fixation**	M00170_C4-dicarboxylic acid cycle, phosphoenolpyruvate carboxykinase type	1302c	1357a	1321bc	1.883 × 10^−5^
	M00172_C4-dicarboxylic acid cycle, NADP—malic enzyme type	3505a	3444b	3445b	0.03007
	M00173_Reductive citrate cycle (Arnon-Buchanan cycle)	10,778b	10,891a	10,850ab	0.004089
	M00374_Dicarboxylate-hydroxybutyrate cycle	7259b	7345a	7333a	0.01178
	M00620_Incomplete reductive citrate cycle, acetyl-CoA => oxoglutarate	2168b	2224a	2231a	0.0007637
**Energy metabolism;;** **Methane metabolism**	M00344_Formaldehyde assimilation, xylulose monophosphate pathway	913b	944a	942ab	0.03158
	M00345_Formaldehyde assimilation, ribulose monophosphate pathway	749b	808a	800a	6.137 × 10^−7^
	M00346_Formaldehyde assimilation, serine pathway	3166b	3234a	3226ab	0.006593
	M00356_Methanogenesis, methanol => methane	22b	26ab	27a	0.03877
	M00358_Coenzyme M biosynthesis	177b	190a	198a	0.0004887
	M00378_F420 biosynthesis	82b	93a	89ab	0.05467
	M00563_Methanogenesis, methylamine/dimethylamine/trimethylamine => methane	465a	434b	464a	3.724 × 10^−6^
**Energy metabolism;** **Nitrogen metabolism**	M00530_Dissimilatory nitrate reduction, nitrate => ammonia	1864a	1823b	1848a	0.01764
**Energy metabolism;** **Sulfur metabolism**	M00176_Assimilatory sulfate reduction, sulfate => H2S	2814a	2741b	2766ab	0.006395
**Glycan metabolism; Glycosaminoglycan metabolis**	M00076_Dermatan sulfate degradation	115b	129a	135a	2.073 × 10^−5^
	M00077_Chondroitin sulfate degradation	105b	118a	123a	6.408 × 10^−5^
	M00078_Heparan sulfate degradation	191b	215a	224a	2.272 × 10^−7^
	M00079_Keratan sulfate degradation	475b	526a	547a	1.002 × 10^−12^
**Glycan metabolism; Lipopolysaccharide metabolism**	M00060_KDO2-lipid A biosynthesis, Raetz pathway, LpxL-LpxM type	3058b	3132a	3124a	0.001684
	M00064_ADP-L-glycero-D-manno-heptose biosynthesis	692b	743a	771a	3.151 × 10^−7^
**Lipid metabolism;** **Fatty acid metabolism**	M00082_Fatty acid biosynthesis, initiation	3785b	3861a	3842ab	0.01467
	M00083_Fatty acid biosynthesis, elongation	9121b	9218a	9214a	0.01719
	M00086_beta-Oxidation, acyl-CoA synthesis	1699b	1743a	1746ab	0.01575
**Lipid metabolism;** **Lipid metabolism**	M00113_Jasmonic acid biosynthesis	428b	454a	438b	0.002276
**Metabolism of cofactors and vitamins;** **Cofactor and vitamin metabolism**	M00116_Menaquinone biosynthesis, chorismate => menaquinol	943b	1026a	977b	1.104 × 10^−10^
	M00117_Ubiquinone biosynthesis, prokaryotes, chorismate => ubiquinone	2772a	2703b	2707ab	0.01037
	M00122_Cobalamin biosynthesis, cobinamide => cobalamin	2143a	2105b	2153a	0.00244
	M00128_Ubiquinone biosynthesis, eukaryotes, 4-hydroxybenzoate => ubiquinone	74a	64b	67ab	0.01119
**Nucleotide metabolism; Purine metabolism**	M00546_Purine degradation, xanthine => urea	2126a	2089b	2125a	0.01079
**Xenobiotics biodegradation;** **Aromatics degradation**	M00537_Xylene degradation, xylene => methylbenzoate	215a	199b	200ab	0.01542
	M00541_Benzoyl-CoA degradation, benzoyl-CoA => 3-hydroxypimeloyl-CoA	59b	67a	67ab	0.02392
	M00548_Benzene degradation, benzene => catechol	27a	20b	21b	0.0002254
	M00551_Benzoate degradation, benzoate => catechol/methylbenzoate => methylcatechol	124a	108b	110b	0.003958
	M00568_Catechol ortho-cleavage, catechol => 3-oxoadipate	445a	421b	433ab	0.01165
	M00569_Catechol meta-cleavage, catechol => acetyl-CoA/4-methylcatechol => propanoyl-CoA	466a	441b	430b	0.001843
	M00637_Anthranilate degradation, anthranilate => catechol	90a	73b	82b	1.726 × 10^−5^

**Table 3:** Topological properties of pairwise functional networks. WTRS: wild tomato related species; TL: tomato landraces; MCC: modern commercial cultivars.

	MCC:WTRS	TL:WTRS	MCC:TL
Number of nodes	133	116	132
Number of edges	1005	1003	1001
Average number of neighbors	15,113	17,293	15,167
Network diameter	6	6	7
Network radius	3	3	4
Characteristic path length	2.542	2.371	2.577
Clustering coefficient	0.000	0.000	0.000
Network density	0.114	0.150	0.116
Network heterogeneity	0.850	0.780	0.869
Network centralization	0.230	0.325	0.309
Connected components	1	1	1

## Data Availability

All raw Illumina sequence data were deposited in the Sequence Read Archive (SRA) service of the European Bioinformatics Institute (EBI) database (BioProject ID: PRJNA693664).

## Supplementary Materials

The following are available online at https://www.mdpi.com/article/10.3390/plants10091942/s1, Table S1: Abundance of predicted functions related to root-associated soil bacteria in the 27 tomato accessions. Table S2: Chemical characteristics of the soil. Table S3: Tab-delimited taxonomy tables.

## References

[1] Reinhold-Hurek B., Bünger W., Burbano C.S., Sabale M., Hurek T. (2015). Roots shaping their microbiome, global hotspots for microbial activity. Annu. Rev. Phytopathol..

[2] Cordovez V., Dini-Andreote F., Carrión V.J., Raaijmakers J.M. (2019). Ecology and Evolution of Plant Microbiomes. Annu. Rev. Microbiol..

[3] Wall D.H., Bardgett R.D., Behan-Pelletier V., Herrick J.E., Jones H., Ritz K., Six J., Strong D.R., van der Putten W.H. (2012). Soil Ecology and Ecosystem Services.

[4] Houlden A., Timms-Wilson T.M., Day M.J., Bailey M.J. (2008). Influence of plant developmental stage on microbial community structure and activity in the rhizosphere of three field crops. FEMS Microbiol. Ecol..

[5] Lei S., Xu X., Cheng Z., Xiong J., Ma R., Zhang L., Yang X., Zhu Y., Zhang B., Tian B. (2019). Analysis of the community composition and bacterial diversity of the rhizosphere microbiome across different plant taxa. Microbiol. Open.

[6] Compant S., Samad A., Faist H., Sessitsch A. (2019). A review on the plant microbiome, ecology; functions; and emerging trends in microbial application. J. Adv. Res..

[7] Liu H., Brettell L.E., Qiu Z., Singh B.K. (2020). Microbiome-mediated stress resistance in plants. Trends Plant. Sci..

[8] Trivedi P., Leach J.E., Tringe S.G., Sa T., Singh B.K. (2020). Plant–microbiome interactions, from community assembly to plant health. Nat. Rev. Microbiol..

[9] Pérez-Jaramillo J.E., Mendes R., Raaijmakers J.M. (2016). Impact of plant domestication on rhizosphere microbiome assembly and functions. Plant. Mol. Biol..

[10] Schmidt J.E., Bowles T.M., Gaudin A.C.M. (2016). Using ancient traits to convert soil health into crop yield, impact of selection on maize root and rhizosphere function. Front. Plant. Sci..

[11] Iannucci A., Fragasso M., Beleggia R., Nigro F., Papa R. (2017). Evolution of the Crop Rhizosphere, Impact of Domestication on Root Exudates in Tetraploid Wheat (*Triticum turgidum* L.). Front. Plant. Sci..

[12] Pérez-Jaramillo J.E., Carrión V.J., de Hollander M., Raaijmakers J.M. (2018). The wild side of plant microbiomes. Microbiome.

[13] Shenton M., Iwamoto C., Kurata N., Ikeo K. (2016). Effect of Wild and Cultivated Rice Genotypes on Rhizosphere Bacterial Community Composition. Rice.

[14] Carrillo J., Ingwell L.L., Li X., Kaplan I. (2019). Domesticated tomatoes are more vulnerable to negative plant–soil feedbacks than their wild relatives. J. Ecol..

[15] Terrazas R.A., Balbirnie-Cumming K., Morris J., Hedley P.E., Russell J., Paterson E., Baggs E.M., Fridman E., Bulgarelli D.A. (2020). A footprint of plant eco-geographic adaptation on the composition of the barley rhizosphere bacterial microbiota. Sci. Rep..

[16] Jia Y., Whalen J.K. (2020). A new perspective on functional redundancy and phylogenetic niche conservatism in soil microbial communities. Pedosphere.

[17] Spor A., Roucou A., Mounier A., Bru D., Breuil M.C., Fort F., Vile D., Roumet P., Philippot L., Violle C. (2020). Domestication-driven changes in plant traits associated with changes in the assembly of the rhizosphere microbiota in tetraploid wheat. Sci. Rep..

[18] Mendes R., Garbeva P., Raaijmakers J.M. (2013). The rhizosphere microbiome, significance of plant beneficial; plant pathogenic; and human pathogenic microorganisms. FEMS Microbiol. Rev..

[19] Martínez-Romero E., Aguirre-Noyola J.L., Taco-Taype N., Martínez-Romero J., Zuñiga-Dávila D. (2020). Plant microbiota modified by plant domestication. Syst. Appl. Microbiol..

[20] Milla R., García-Palacios P., Matesanz S. (2017). Looking at past domestication to secure ecosystem services of future croplands. J. Ecol..

[21] Stevenson F.J. (1994). Humus Chemistry, Genesis, Composition, Reactions.

[22] Zhang Y., Yue D., Ma H. (2015). Darkening mechanism and kinetics of humification process in catechol-Maillard system. Chemosphere.

[23] Tiessen H., Cuevas E., Chacon P. (1994). The role of soil organic matter in sustaining soil fertility. Nature.

[24] Gougoulias C., Clark J.M., Shaw L.J. (2014). The role of soil microbes in the global carbon cycle, tracking the below-ground microbial processing of plant-derived carbon for manipulating carbon dynamics in agricultural systems. J. Sci. Food Agric..

[25] Six J., Frey S.D., Thiet R.K., Batten K.M. (2006). Bacterial and Fungal Contributions to Carbon Sequestration in Agroecosystems. Soil Sci. Soc. Am. J..

[26] Pérez-Jaramillo J.E., de Hollander M., Ramírez C.A., Mendes R., Raaijmakers J.M., Carrión V.J. (2019). Deciphering rhizosphere microbiome assembly of wild and modern common bean (*Phaseolus vulgaris*) in native and agricultural soils from Colombia. Microbiome.

[27] Vries F., Wallenstein M.D. (2017). Below-ground connections underlying above-ground food production: A framework for optimising ecological connections in the rhizosphere. J. Ecol..

[28] Cavicchioli R., Ripple W.J., Timmis K.N., Azam F., Bakken L.R., Baylis M., Behrenfeld M.J., Boetius A., Boyd P.W., Classen A.T. (2019). Scientists’ warning to humanity, microorganisms and climate change. Nat. Rev. Microbiol..

[29] Lynn T.M., Ge T., Yuan H., Wei X., Wu X., Xiao K., Kumaresan D., Yu S.S., Wu J., Whiteley A.S. (2017). Soil Carbon-Fixation Rates and Associated Bacterial Diversity and Abundance in Three Natural Ecosystems. Microb. Ecol..

[30] de Carvalho C., Caramujo M.J. (2018). The Various Roles of Fatty Acids. Molecules.

[31] Pieterse C.M., Leon-Reyes A., Van der Ent S., Van Wees S.C. (2009). Networking by small-molecule hormones in plant immunity. Nat. Chem. Biol..

[32] Bari R., Jones J.D. (2009). Role of plant hormones in plant defence responses. Plant. Mol. Biol..

[33] Kenrick P., Crane P. (1997). The origin and early evolution of plants on land. Nature.

[34] Pozo M.J., López-Ráez J.A., Azcón-Aguilar C., García-Garrido J.M. (2015). Phytohormones as integrators of environmental signals in the regulation of mycorrhizal symbioses. New Phytol..

[35] Gruden K., Lidoy J., Petek M., Podpečan V., Flors V., Papadopoulou K.K., Pappas M.L., Martinez-Medina A., Bejarano E., Biere A. (2020). Ménage à Trois, Unraveling the Mechanisms Regulating Plant-Microbe-Arthropod Interactions. Trends Plant. Sci..

[36] Eng F., Marin J.E., Zienkiewicz K., Gutiérrez-Rojas M., Favela-Torres E., Feussner I. (2021). Jasmonic acid biosynthesis by fungi, derivatives; first evidence on biochemical pathways and culture conditions for production. PeerJ.

[37] Dagorn A., Chapalain A., Mijouin L., Hillion M., Duclairoir-Poc C., Chevalier S., Taupin L., Orange N., Feuilloley M.G. (2013). Effect of GABA, a bacterial metabolite; on *Pseudomonas fluorescens* surface properties and cytotoxicity. Int. J. Mol. Sci..

[38] Somorin Y., Abram F., Brennan F., O’Byrne C. (2016). The General Stress Response Is Conserved in Long-Term Soil-Persistent Strains of Escherichia coli. Appl. Environ. Microbiol..

[39] Li L., Wang Z., He P., Ma S., Du J., Jiang R. (2016). Construction and Analysis of Functional Networks in the Gut Microbiome of Type 2 Diabetes Patients. Genom. Proteom. Bioinform..

[40] FAO (2015). World Reference Base for Soil Resources 2014: International Soil Classification System for Naming Soils and Creating Legends for Soil Maps.

[41] Ferrero V., Baeten L., Blanco-Sanchez L., Planello R., Diaz-Pendon J.A., Rodríguez-Echeverría S., Haegeman A., de la Peña E. (2020). Complex patterns in tolerance and resistance to pests and diseases underpin the domestication of tomato. New Phytol..

[42] Takahashi S., Tomita J., Nishioka K., Hisada T., Nishijima M. (2014). Development of a prokaryotic universal primer for simultaneous analysis of Bacteria and Archaea using next-generation sequencing. PLoS ONE.

[43] Lundberg D.S., Yourstone S., Mieczkowski P., Jones C.D., Dangl J.L. (2013). Practical innovations for high-throughput amplicon sequencing. Nat. Methods.

[44] Vetrovský T., Baldrian P., Morais D. (2018). SEED 2, a user-friendly platform for amplicon high-throughput sequencing data analyses. Bioinformatics.

[45] Edgar R.C. (2013). UPARSE, highly accurate OTU sequences from microbial amplicon reads. Nat. Methods.

[46] Schloss P.D., Westcott S.L., Ryabin T., Hall J.R., Hartmann M., Hollister E.B., Lesniewski R.A., Oakley B.B., Parks D.H., Robinson C.J. (2009). Introducing mothur, open-source; platform-independent; community-supported software for describing and comparing microbial communities. Appl. Environ. Microbiol..

[47] Quast C., Pruesse E., Yilmaz P., Gerken J., Schweer T., Yarza P., Peplies J., Glöckner F.O. (2013). The SILVA ribosomal RNA gene database project, improved data processing and web-based tools. Nucleic Acids Res..

[48] Chong J., Liu P., Zhou G., Xia J. (2020). Using MicrobiomeAnalyst for comprehensive statistical; functional; and meta-analysis of microbiome data. Nat. Protoc..

[49] Dhariwal A., Chong J., Habib S., King I.L., Agellon L.B., Xia J. (2017). MicrobiomeAnalyst, a web-based tool for comprehensive statistical; visual and meta-analysis of microbiome data. Nucleic Acids Res..

[50] Aßhauer K.P., Wemheuer B., Daniel R., Meinicke P. (2015). Tax4Fun, predicting functional profiles from metagenomic 16S rRNA data. Bioinformatics.

[51] Breiman L. (2001). Random forests. Mach. Learn..

[52] Segata N., Izard J., Waldron L., Gevers D. (2011). Metagenomic biomarker discovery and explanation. Genome Biol..

[53] Faust K., Raes J. (2016). CoNet app, inference of biological association networks using Cytoscape. F1000Research.

[54] Shannon P., Markiel A., Ozier O., Baliga N.S., Wang J.T., Ramage D., Amin N., Schwikowski B., Ideker T. (2003). Cytoscape, a software environment for integrated models of biomolecular interaction networks. Genome Res..

[55] Weiss S., Van Treuren W., Lozupone C., Faust K., Friedman J., Deng Y., Xia L.C., Xu Z.Z., Ursell L., Alm E.J. (2016). Correlation detection strategies in microbial data sets vary widely in sensitivity and precision. ISME J..

[56] Bader G.D., Hogue C.W. (2003). An automated method for finding molecular complexes in large protein interaction networks. BMC Bioinform..

[57] Fox J., Weisberg S. (2019). An R Companion to Applied Regression.

[58] Lenth R.V. (2016). Least-squares means, The R package lsmeans. J. Stat. Soft.

[59] R Development Core Team (2017). R, A Language and Environment for Statistical Computing.

[60] RStudio Team (2016). RStudio, Integrated Development Environment for R.

