# Supplementary material for: Tomato Domestication Affects Potential Functional Molecular Pathways of Root-Associated Soil Bacteria
Source: Plants (Basel). 2021 Sep 17;10(9):1942. doi: 10.3390/plants10091942 (PMC8472556; doi:10.3390/plants10091942)
Supplement: Supplementary file 1 [file plants-10-01942-s001.zip › Table S2.pdf]

Table S2: TN: Total Nitrogen (%), TOC: Total Organic Carbon (%), and elemental analysis of nutrients (ppm) in soil

TN	TOC	Al	As	Ca	Cd
0.303±0.008	1.76±0.073	40938±1244	31±0.613	9226±230	2.22±0.022
**Co**	**Cr**	**Cu**	**Fe**	**K**	**Li**
22±0.250	54±1.10	39±0.264	33233±298	10525±342	29±0.403
**Mg**	**Mn**	**Na**	**Ni**	**P**	**Pb**
8086±78	875±20	0.046±0.002	46±0.884	995±41	24±0.585
**S**	**Si**	**Sr**	**Ti**	**V**	**Zn**
432±11	3718±110	53±0.919	1326±27	69±1.62	102±3.37
